# Enhanced MDM2 Oncoprotein Expression in Soft Tissue Sarcoma: Several Possible Regulatory Mechanisms

**DOI:** 10.1080/13577149778443

**Published:** 1997-03

**Authors:** Raphael E. Pollock, Aiquing Lang, Adel K. El-Naggar, Robert Radinsky, Mien Chie Hung

**Affiliations:** 1 Department of Surgical Oncology MD Anderson Cancer Center 1515 Holcombe Blvd, Box 106 University of Texas Houston TX 77030 USA; 2 Department of Tumor Biology MD Anderson Cancer Center 1515 Holcombe Blvd, Box 106 University of Texas Houston TX 77030 USA; 3 Department of Pathology MD Anderson Cancer Center University of Texas USA; 4 Department of Cell Biology MD Anderson Cancer Center University of Texas USA

## Abstract

*Purpose. MDM2* is an oncogene whose protein product may promote tumorigenesis by blocking wild-type p53 tumor suppressor mediated G _0_/G_1_ cell cycle arrest, thereby inhibiting repair of damaged DNA prior to cell division. While *MDM2* DNA amplification is frequently observed in human sarcoma, the mechanisms linking this amplification to MDM2 oncoprotein over-production as well as its functional significance have not been well characterized in patients with soft tissue sarcoma.

*Methods*. A tissue bank of resected soft tissue sarcomas and autologous normal tissues was assembled; all specimens were snap frozen within 15 min of resection. DNA and RNA were extracted from tissues using isoamyl alcohol and phenol chloroform extraction methods, respectively; cell lysates were prepared using PBSTDS lysis buffer. DNA and mRNA were confirmed as being non-degraded and were then examined for *MDM2* DNA amplification (Southern blots) and mRNA over-expression (Northern blots) using actin (DNA) and glyceraldehyde-3-phosphate dehydrogenase (mRNA) as loading controls. The MDM2 protein was examined on Western blots using the MDM2-specific monoclonal antibody IF2 (Oncogene Science, Inc). The presence of p53 DNA and expression of p53 mRNA was examined by rehybridizing the Southern and Northern filters using a p53-specific cDNA probe.

*Results*. Soft tissue sarcomas and autologous normal tissues were screened for *MDM2* DNA amplification, which was detected in 10 of 30 tumors screened. After screening, there was sufficient biomaterials from six specimens for subsequent Northern and Western analysis to see whether *MDM2* gene amplification correlated with over-expression of MDM2 mRNA and MDM2 protein. In addition, we examined whether other mechanisms may lead to over-expression of the MDM2 oncoprotein. Several possible mechanisms of MDM2 oncoprotein over-expression were identified. These most commonly included *MDM2* DNA amplification, MDM2 mRNA over-expression and MDM2 oncoprotein over-expression. However, some soft tissue sarcoma patient specimens had no evidence of MDM2 mRNA over-expression yet had MDM2 oncoprotein over-production in the tumor relative to autologous normal tissue, implying possible post-transcriptional regulation. Of functional relevance, MDM2 oncoprotein over-production by tumors was associated with large decreases in the percentage of cells in the _0_/G_1_ cell cycle interface compared with autologous normal tissue cells.

*Discussion*. It is likely that there are multiple mechanisms underlying human soft tissue sarcoma MDM2 oncoprotein over-production. Consequently, strategies that decrease MDM2 over-production, such as transcriptional repression to inhibit MDM2 promoter activity or RNA antisense approaches, may ultimately offer the best therapeutic efficacy.


*Sarcoma* (1997) **1**, 23–29

ORIGINAL ARTICLE

### Enhanced MDM2 oncoprotein expression in soft tissue sarcoma: several possible regulatory mechanisms

RAPHAEL E. POLLOCK,[1,2] AIQUING LANG,[1] ADEL K. EL-NAGGAR,[3] ROBERT RADINSKY[4] & MIEN CHIE HUNG[2]

*Departments of [1]Surgical Oncology, [2]Tumor Biology, [3]Pathology and [4]Cell Biology, MD Anderson Cancer Center, University of Texas, USA*

**Abstract**

*Purpose.* *MDM2* is an oncogene whose protein product may promote tumorigenesis by blocking wild-type p53 tumor suppressor mediated $G_0/G_1$ cell cycle arrest, thereby inhibiting repair of damaged DNA prior to cell division. While *MDM2* DNA amplification is frequently observed in human sarcoma, the mechanisms linking this amplification to MDM2 oncoprotein over-production as well as its functional significance have not been well characterized in patients with soft tissue sarcoma.

*Methods.* A tissue bank of resected soft tissue sarcomas and autologous normal tissues was assembled; all specimens were snap frozen within 15 min of resection. DNA and RNA were extracted from tissues using isoamyl alcohol and phenol chloroform extraction methods, respectively; cell lysates were prepared using PBSTDS lysis buffer. DNA and mRNA were confirmed as being non-degraded and were then examined for *MDM2* DNA amplification (Southern blots) and mRNA over-expression (Northern blots) using actin (DNA) and glyceraldehyde-3-phosphate dehydrogenase (mRNA) as loading controls. The MDM2 protein was examined on Western blots using the MDM2-specific monoclonal antibody IF2 (Oncogene Science, Inc). The presence of p53 DNA and expression of p53 mRNA was examined by rehybridizing the Southern and Northern filters using a p53-specific cDNA probe.

*Results.* Soft tissue sarcomas and autologous normal tissues were screened for *MDM2* DNA amplification, which was detected in 10 of 30 tumors screened. After screening, there was sufficient biomaterials from six specimens for subsequent Northern and Western analysis to see whether *MDM2* gene amplification correlated with over-expression of MDM2 mRNA and MDM2 protein. In addition, we examined whether other mechanisms may lead to over-expression of the MDM2 oncoprotein. Several possible mechanisms of MDM2 oncoprotein over-expression were identified. These most commonly included *MDM2* DNA amplification, MDM2 mRNA over-expression and MDM2 oncoprotein over-expression. However, some soft tissue sarcoma patient specimens had no evidence of MDM2 mRNA over-expression yet had MDM2 oncoprotein over-production in the tumor relative to autologous normal tissue, implying possible post-transcriptional regulation. Of functional relevance, MDM2 oncoprotein over-production by tumors was associated with large decreases in the percentage of cells in the $G_0/G_1$ cell cycle interface compared with autologous normal tissue cells.

*Discussion.* It is likely that there are multiple mechanisms underlying human soft tissue sarcoma MDM2 oncoprotein over-production. Consequently, strategies that decrease MDM2 over-production, such as transcriptional repression to inhibit MDM2 promoter activity or RNA antisense approaches, may ultimately offer the best therapeutic efficacy.

**Key words:** *soft tissue sarcoma, MDM2, oncogene amplification.*

### Introduction

The *MDM2* oncogene codes for a 90-kDa protein may be involved in regulation of tumor proliferation via suppression of wild-type (wt) p53 mediated $G_1$ cell cycle checkpoint control.[1,2] MDM2 can overcome wt p53 suppression of transformed cell growth,[3] most likely by forming a complex with wt p53[4] and thereby concealing the wt p53 activation domain.[5] Experimentally induced DNA damage increases levels of MDM2 mRNA in cells expressing wt p53;[6] wt p53 may transcriptionally activate the *MDM2* gene.[7,8] These interactions suggest an autoregulatory feedback loop in which wt p53 protein regulates the *MDM2* gene at the transcriptional level and MDM2 protein regulates wt p53 protein at the activity level.[9,10] This autoregulatory network as elucidated in the above cell line experimentation suggests that the coordinate presence of mutated p53 and MDM2 would be redundant in suppressing G1

Correspondence to: R. E. Pollock, University of Texas, MD Anderson Cancer Center, 1515 Holcombe Blvd, Box 106, Houston, TX 77030, USA. Tel: +1 713 792 6928; Fax: +1 713 792 0722; E-mail: raphael-pollock@surg.mdacc.tmc.edu.

1357-714X/97/010023–07 ©1997 Journals Oxford Ltd checkpoint control.[9] Consistent with this, most initial tumor tissue observations have failed to detect *MDM2* gene amplification in the presence of mutated p53 gene.[9]

MDM2 oncoprotein inactivation of tumor suppressor gene function may also alternatively involve physical and functional interaction with RB, the protein product of the retinoblastoma tumour suppressor gene, thereby inhibiting RB-mediated tumor growth regulation.[11] Moreover, the MDM2 oncoprotein may not only release a tumor proliferative control by blocking p53 and RB, but may also positively augment tumor proliferation by stimulating the cell cycle S-phase inducing transcription factors E2F1/DP1.[12] Studies in MDM2-deficient mice have provided important insights regarding the interaction of MDM2 and p53 *in vivo*.[13,14] *MDM2* null mice are not viable; however, *MDM2* null lethality is avoidable in mice that are also p53 null, suggesting a critical *in vivo* role of MDM2 as a negative regulator of p53 function in normal development.[14]

Mechanisms underlying the production of MDM2 protein in tumor tissue have not yet been fully characterized. However, *MDM2* gene amplification has been described in a variety of tumors including carcinoma of the breast,[15] glioma,[16–18] pancreatic adenocarcinoma,[19,20] osteosarcoma,[21] urothelial carcinomas,[22,23] some leukemias,[24] Hodgkin's[25] and non-Hodgkin's lymphomas,[26] several gynecologic malignancies,[27] neuroblastoma[28] and germ cell tumors,[29] and as an especially frequent finding in soft tissue sarcoma.[30]

In light of these considerations, we sought to analyze MDM2 in soft tissue sarcoma and autologous normal tissue at the DNA, RNA and protein levels to delineate possible mechanisms underlying sarcoma over-production of MDM2 oncoprotein. Our studies suggest several patterns of regulation including *MDM2* gene amplification, possible transcriptional and post-transcriptional regulation, and coordinate presence of *MDM2* gene amplification with possible p53 gene mutation, a finding not anticipated by the *MDM2*–p53 autoregulatory loop hypothesis.

### Patients and methods

*Tissue acquisition and preservation*

Soft tissue sarcoma, autologous normal skeletal muscle resected as part of the surgical specimens and autologous peripheral blood lymphocytes were retrieved, fast-frozen in liquid nitrogen, and stored in a $-140^{\circ}$C freezer for subsequent usage. These tissues were acquired as part of a clinical research protocol approved by the University of Texas MD Anderson Cancer Center Surveillance Committee (Institutional Review Board).

*DNA analysis*

High molecular weight DNA from normal and tumor samples was extracted by proteinase K digestion and phenol/chloroform extraction. A 2.2-kb human *MDM2* fragment and a 1.8-kb human p53 fragment were used as probes in Southern blot analyses. A $\beta$-actin probe was used as an internal loading control. Both the human *MDM2* and p53 plasmids were provided as a kind gift by Dr B. Vogelstein. For the Southern blot analysis, 20 $\mu$g of genomic DNA were digested with *Eco*RI restriction endonuclease overnight and then electrophoretically separated on 0.8% agarose gel and blotted to nylon membranes. The membranes were prehybridized with $1\times$Church buffer at $65^{\circ}$C for 1 h and hybridized with the *MDM2* DNA probe that had been labeled at $65^{\circ}$C overnight with [$^{32}$P]dCTP using a random primer DNA labeling kit (Boeringer Mannheim Corp., Indianapolis, IN, USA). Membranes were then washed and subjected to autoradiography at $-80^{\circ}$C for 4–20 h using intensifying screens. The same membranes were then washed twice with 0.1% sodium dodecyl sulfide followed by 0.1X SSC at $100^{\circ}$C for 20 min for each wash and then rehybridized with the human p53 and $\beta$-actin probes.

*mRNA analysis*

Total cellular RNA was extracted directly from snap frozen tumor specimens using a guanidinium thiocyanate–hot phenol method as described previously.[31] For Northern blot analyses, Poly(A$^{+}$) RNA was prepared by oligo(dT)-cellulose chromatography, fractionated on 1% denaturing formaldehyde–agarose gels, electrotransferred at 0.6 A to a GeneScreen nylon membrane (DuPont Co., Boston, MA, USA) and UV cross-linked with 120 000 $\mu$J cm$^{-2}$ using a UV Stratalinker 1800 (Stratagene, La Jolla, CA, USA). Filters were washed two or three times at $60^{\circ}$C with 30 mM NaCl-3 mM sodium citrate (pH 7.2)–0.1% $NaDodSO_4$ (w/v). A glyceraldehyde-3-phosphate dehydrogenase (GAPDH) probe was used as a loading control. The cDNA probes used were a 2.2-kb restriction endonuclease fragment from the plasmid corresponding to the full length *MDM2* human cDNA.

*Cellular protein analysis*

Western blot analyses of autologous sarcoma and normal tissues were performed as previously described.[32] The nitrocellulose membrane was blocked with 2% non-fat milk in TPBS buffer (0.05% Tween-20 in phosphate-buffered salup (PBS) buffer) for 1 h and then incubated with 1 $\mu$g ml$^{-1}$ of MDM2-specific monoclonal antibody Ab IF2 (Oncogene Science, Inc., Uniondale, NY, USA).

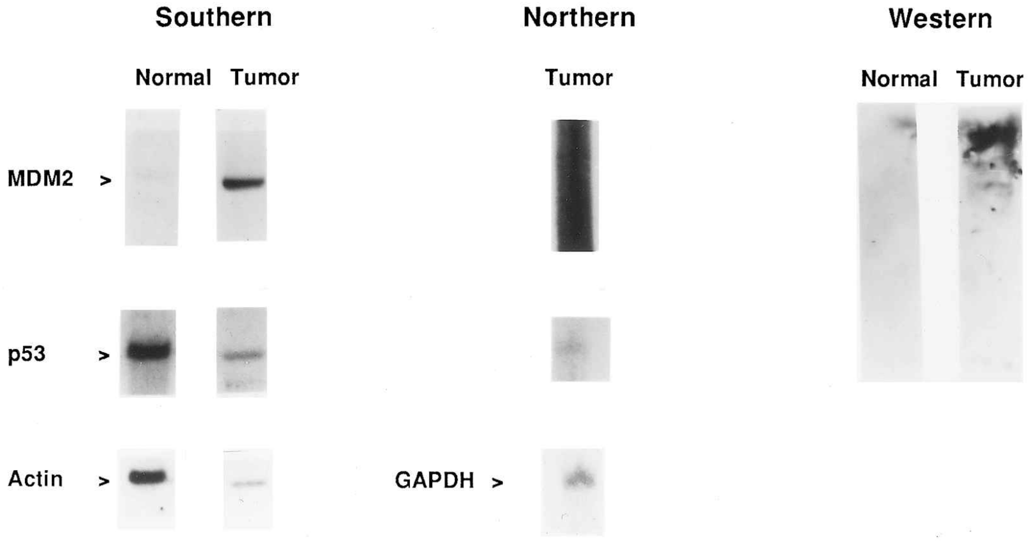

**Fig. 1.** *Standard pattern of MDM2 oncoprotein over-production. DNA analysis demonstrates* MDM2 *gene amplification. There is MDM2 mRNA expression and increased MDM2 oncoprotein production relative to autologous normal tissue.*

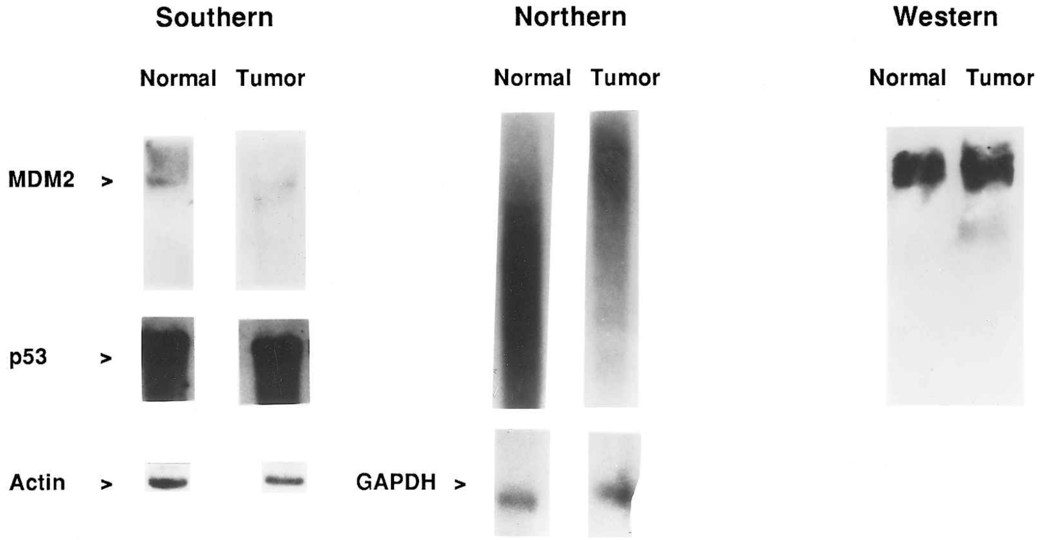

**Fig. 2.** *Lack of MDM2 oncoprotein over-production. There is no detectable* MDM2 *gene amplification or mRNA over-expression. MDM2 oncoprotein production is equivalent in sarcoma and autologous normal tissue.*

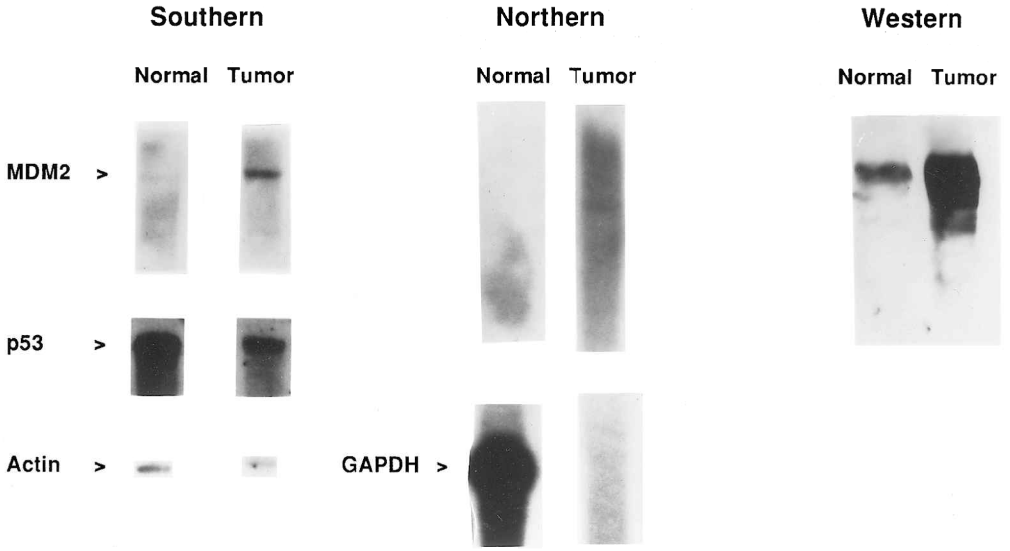

**Fig. 3.** *MDM2 oncoprotein over-production: possible post-transcriptional regulation. While there is clear* MDM2 *DNA amplification in the tumor compared with autologous normal tissue, MDM2 mRNA does not appear to be over-expressed in the tumor. However, there is obvious increased tumor MDM2 oncoprotein over-production.*

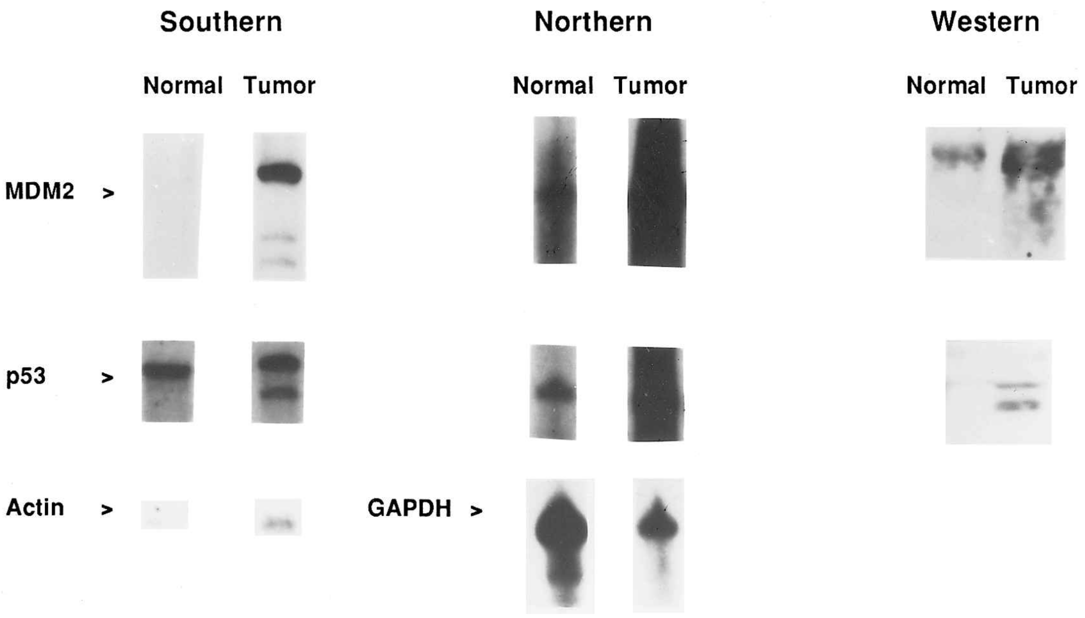

**Fig. 4.** *MDM2 oncoprotein over-production: possible p53 mutations. The Southern analysis demonstrates* MDM2 *DNA amplification in tumor as well as a possible coexisting p53 mutation. Over-expressed mRNA and tumor over-production of MDM2 oncoprotein are also observable.*

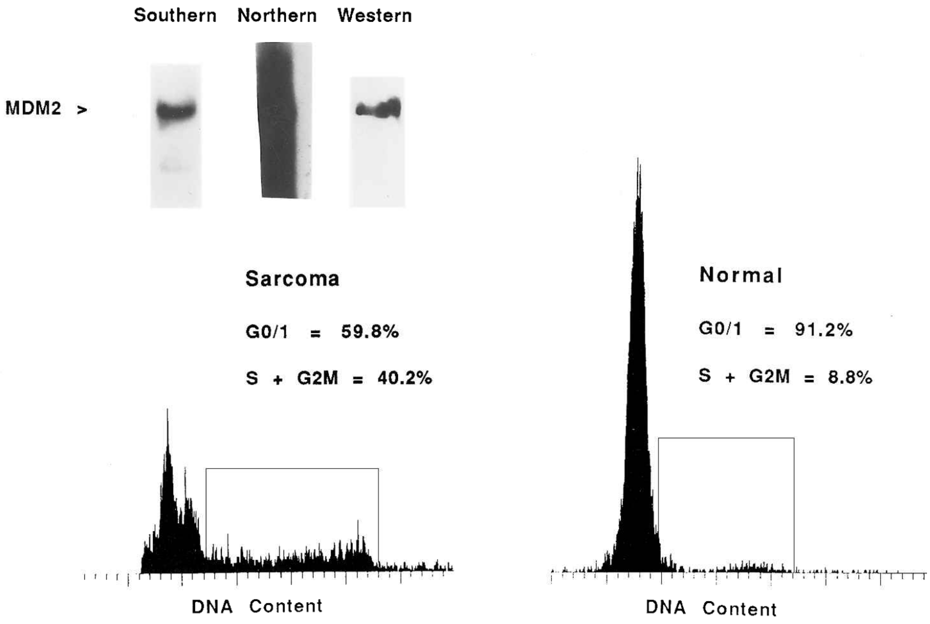

**Fig. 5.** *Dysregulated cell-cycle kinetics in a sarcoma with MDM2 oncoprotein over-production. The standard pattern of MDM2 oncoprotein over-production is observed as per Fig. 1. The percentage of cells in the $S + G_2M$ phase is increased more than four-fold in the sarcoma relative to the autologous normal tissue.*

*Flow cytometry*

Disaggregated cells were adjusted to $1.0 \times 10^6$ cells/ml for flow cytometric analysis of DNA content in fresh tissues. A cytospin preparation was evaluated for preparation quality and nuclear integrity. Cells were subsequently stained with acridine orange using the standard two-step method. Ploidy status was defined by the DNA index, which represents the ratio of the relative $G_0/G_1$ diploid peak. Near-diploid (hypodiploid or hyperdiploid) DNA was determined after mixing a test sample with lymphocyte controls.

### Results

An initial screen of 30 resected soft tissue sarcomas identified 10 tumors with *MDM2* amplication at the DNA level. In several patients (predominantly liposarcoma) there was sufficient sarcoma as well as autologous normal tissue to support a more extensive analysis at the DNA, mRNA and cellular protein levels. Figure 1 is representative of the typical pattern of MDM2 protein over-production in sarcoma relative to that in autologous normal tissue: *MDM2* DNA amplification in tumor (Southern blot), abundant MDM2 mRNA (over) expression in the tumor (Northern blot) and MDM2 protein over-expression by sarcoma (Western blot) compared to the autologous normal tissue. In contrast, Fig. 2 is representative of the more typical pattern observed at the initial tumor screening: no sarcoma *MDM2* DNA amplification, and equivalent levels of MDM2 mRNA expression with comparable levels of MDM2 protein production in both the sarcoma and autologous normal tissues.

Figure 3 illustrates an alternative possible mechanism of MDM2 protein over-production by tumor. While there is relative sarcoma *MDM2* DNA amplification, there is minimal if any detectable MDM2 mRNA over-expression in the tumor. However, there is markedly increased MDM2 protein production in the tumor relative to that in autologous normal tissue, implying a possible *MDM2* post-transcriptional level of regulatory control.

MDM2 may interact with p53 in an autoregulatory loop control process such that p53 protein regulates the *MDM2* gene at the level of transcription, and MDM2 protein regulates the p53 protein at the level of its activity.[9] Because this hypothesized feedback loop regulates both the activity of the p53 protein as well as the expression of the *MDM2* gene, it would be redundant for a tumor to contain both *MDM2* gene amplification and p53 gene mutations. However, Fig. 4 illustrates the possibility that these two alterations may coexist within a single sarcoma. As depicted, there is *MDM2* DNA amplification in the sarcoma relative to that seen in autologous normal tissue. In addition, there is a possible p53 mutation detectable in the sarcoma that is not detected in the normal tissue. Both MDM2 and p53 mRNA transcripts were present, and there was relative MDM2 and p53 protein over-production in the sarcoma tissues compared to the autologous control tissues.

Lastly, we analyzed the potential functional significance of MDM2 protein over-production given that *MDM2* blockade of wt p53 leads to loss of $G_0/G_1$ cell cycle arrest and a concomitant increase in the percentage of cells in the $S + G_2M$ phase of the cell cycle. Figure 5 depicts flow cytometry histograms for a representative sarcoma and autologous normal tissue where there was concomitant sarcoma *MDM2* DNA amplification and MDM2 protein over-production. Cell cycle dysregulation is suggested in that the normal tissue percentage of cells in the $G_0/G_1$ cell cycle phase was 91.2% and the $S + G_2M$ percentage was 8.8%, whereas the comparable percentages in the sarcoma were 59.8% and 40.2%, respectively.

### Discussion

While *MDM2* gene amplification appears to be a relatively common event in soft tissue sarcoma and several other solid malignancies, the spectrum of involvement apparently does not include medulloblastoma,[33] melanoma,[34] Ewing's sarcoma,[35] cervical carcinoma,[36] myelodysplastic syndromes,[37] Wilms' tumor[38] and esophageal carcinoma.[39] However, many studies have considered only *MDM2* gene amplifications (Southern analysis) or, alternatively, MDM2 protein production. This strategy may miss possible translational or post-transcriptional regulatory mechanisms; therefore, the spectrum of tumor involvement may perhaps be broader than has been initially described.

In keeping with this possibility, Landers *et al.* recently described experiments using two different choriocarcinoma cell lines in which elevated MDM2 protein levels were not associated with gene amplification or over-expression of MDM2 mRNA.[40] Instead, a post-transcriptional regulatory mechanism was shown to be responsible for enhanced translation. Olson *et al.* demonstrated four other MDM2 protein forms (p85, p76, p74 and p58–p57) in addition to p90 that were detected in a spontaneously transformed BALB/c mouse 3T3 cell line using monoclonal and polyclonal antibodies generated against murine and human MDM2 proteins.[41] It is possible that these alternative proteins could have arisen from various spliced mRNA forms of the *MDM2* gene or by post-translational MDM2 protein alterations.[41]

Working with breast carcinoma cell lines, Sheikh *et al.* demonstrated that the high constitutive MDM2 mRNA levels observed in estrogen receptor positive cells was the result of differential regulation of the *MDM2* gene at the transcriptional and/or post-transcriptional level.[15] Similar post-transcriptional regulatory mechanisms were also identified in studies involving breast carcinoma cell lines conducted by Gudas *et al.*[42] However, to date, MDM2 post-transcriptional regulation has not been analyzed in soft tissue sarcoma, perhaps because of the relative rarity of the disease and the difficulties in extracting mRNA from sarcoma tissues.[30,43]

Several studies have focused on the issue of concomitant *MDM2* gene amplification and p53 gene mutation in soft tissue sarcoma. Leach *et al.* analyzed 24 soft tissue sarcomas, identifying eight tumors with p53 mutations and a non-overlapping subset of another eight sarcomas with *MDM2* gene amplification (DNA analysis).[44] It was concluded that p53 and *MDM2* genetic alterations were alternative mechanisms for inactivating the same regulatory pathway and that double mutations in both p53 and *MDM2* were redundant and provided no selective advantage over mutation in only one of the two genes.

Cordon-Cardo *et al.* examined a larger selection of soft tissue sarcomas using Southern analysis and immunohistochemical analysis.[45] Seventy-six of 211 sarcomas had abnormally high levels of MDM2 protein; 56 of these 211 tumors over-expressed p53 proteins. Twenty-two of these 211 sarcomas had abnormally high levels of both p53 and MDM2

proteins. These 22 patients had significantly impaired survival relative to the other individuals studied. However, these immunohistochemical determinations apparently did not include a direct comparison of p53 and MDM2 protein status that incorporated autologous normal control tissues. Because of possible differences between autologous normal and sarcoma tissues, as we have detected in the *MDM2* Western analyses here, it is difficult to conclude that the presence of immunohistochemical positive staining for both p53 and *MDM2* in the same sarcoma sample means that there is a coexisting double mutation of these respective genes.

In this same analysis, over-expression of either p53 or MDM2 protein on immunohistochemistry did not always correlate with *MDM2* gene amplification or p53 gene mutation as determined by DNA analysis. While 11 of 73 sarcomas had *MDM2* amplification interpreted as at least 5–35 fold gene copy excess, only six of these 11 tumors had increased MDM2 protein. In contrast, 17 of 62 sarcomas with increased MDM2 protein lacked detectable *MDM2* gene amplification.

These findings suggest that there might be additional *MDM2* gene mutations not detected by the Southern analysis, or that the immunohistochemical analysis did not detect the presence of one or another of the alternative MDM2 protein forms. As an additional possibility, post-transcriptional or post-translational regulation may be operative, as is suggested in Fig. 3, or perhaps MDM2 mRNA levels were under-expressed. These latter possibilities might be resolvable using an MDM2 mRNA analytic strategy.

Alternatively, since *MDM2* over-expression is sufficient for inactivation of wt p53 function, the possible double mutation of both p53 and *MDM2* genes in the sarcoma depicted in Fig. 4 and the analysis of Cordon-Cardo *et al.* suggests that there might be a 'gain of function' in the development of sarcoma. Additional studies, possibly utilizing co-transfection strategies, will be required to address this issue.

Florenes *et al.* examined 68 sarcomas, 26 human sarcoma xenografts and two sarcoma cell lines for *MDM2* amplification and p53 mutations in exons 5, 7 or 8, as well as p53 and MDM2 mRNA over-expression.[46] They observed amplification of the *MDM2* gene in 10 tumors, nine of which also had MDM2 mRNA over-expression. However, MDM2 mRNA was also over-expressed in an additional three sarcomas that lacked *MDM2* gene amplification. None of the tumors with *MDM2* gene amplification had mutations detected in their respective p53 genes. The presence of MDM2 or p53 protein product production was not examined in this study. Taken together, these reports point to the importance of experimental schema that consider MDM2 and p53 status on the DNA, RNA and protein levels in both sarcomas and paired autologous tissues.

Therapeutic strategies to blunt the functional impact of *MDM2* gene amplification that leads to over-production of MDM2 oncoprotein will need to account for the possibility of multiple regulatory mechanisms, multiple oncoprotein products, and the yet to be established potential interplay between simultaneous *MDM2* gene amplification and p53 gene mutations. Transcriptional regulation, RNA antisense approaches or even augmentation of wt p53 cell cycle regulatory function in *MDM2* amplified tumors may prove to be feasible and of future therapeutic benefit for patients burdened by soft tissue sarcoma.

### Acknowledgements

We would like to acknowledge the excellent secretarial support of Elizabeth B. Hardham. This research was supported by NCI NIH RO1 CA67802 (REP), NCI NIH R29 CA67952 (RR), the Sid Richardson Foundation (RR) and the NCI NIH Cancer Center Core Grant CA16672.

### References

1 Chen CY, Oliner JD, Zhan Q, *et al.* Interactions between p53 and MDM2 in a mammalian cell cycle checkpoint pathway. *Proc Natl Acad Sci* 1994; 91:2684–8.

2 Fakharzadeh SS, Trusko SP, George DL. Tumorigenic potential associated with enhanced expression of a gene that is amplified in a mouse tumor. *EMBO J* 1991; 10:1565–9.

3 Finlay CA. The mdm-2 oncogene can overcome wild-type p53 suppression of transformed cell growth. *Mol Cell Biol* 1993; 13:301–6.

4 Barak Y, Oren M. Enhanced binding of a 95 kDa protein to p53 in cells undergoing p53-mediated growth arrest. *EMBO J* 1992; 11:2115–21.

5 Momand J, Zanbetti GP, Olson DC, *et al.* The mdm-2 oncogene product forms a complex with p53 protein and inhibits p53-mediated transactivation. *Cell* 1992; 69:1237–45.

6 Price BD, Park SJ. DNA damage increases the levels of MDM2 messenger RNA in wt p53 human cells. *Cancer Res* 1994; 54:896–9.

7 Juven T, Barak Y, Zauberman A, *et al.* Wild type p53 can mediate sequence-specific transactivation of an internal promoter within the mdm2 gene. *Oncogene* 1993; 8:3411–16.

8 Barak Y, Juven T, Haffner R, *et al.* mdm2 expression is induced by wild type p53 activity. *EMBO J* 1993; 12:461–8.

9 Wu X, Bayle H, Olson D, *et al.* The p-53–mdm-2 autoregulatory feedback loop. *Genes and Dev* 1993; 7:1126–32.

10 Otto A, Deppert W. Upregulation of mdm-2 expression in meth A tumor cells tolerating wild-type p53. *Oncogene* 1993; 8:2591–603.

11 Xiao Z, Chen J, Levine AJ, *et al.* Interaction between the retinoblastoma protein and the oncoprotein MDM2. *Nature* 1995; 375:694–8.

12 Martin K, Trouche D, Hagemeier C, *et al.* Stimulation of E2F1/DP1 transcriptional activity by MDM2 oncoprotein. *Nature* 1995; 375:691–4.

13 Jones SN, Roe AE, Donehower LA, *et al.* Rescue of embryonic lethality in Mdm2-deficient mice by absence of p53. *Nature* 1995; 378:206–8.

14 Montes de Oca Luna R, Wagner DS, Lozano G. Rescue of early embryonic lethality in mdm2-deficient mice by deletion of p53. *Nature* 1995; 378:203–6.

15 Sheikh MS, Shao Z-M, Hussain A, *et al.* The p53-binding protein MDM2 gene is differentially expressed in human breast carcinoma. *Cancer Res* 1993; 53:3226–8.

16 Reigenberger G, Reifenberger J, Ichimura K, *et al.* Amplification of multiple genes from chromosomal region 12q13–14 in human malignant gliomas: preliminary mapping of the amplicons shows preferential involvement of CDK4, SAS and MDM2. *Cancer Res* 1994; 54:4299–303.

17 Reigenberger G, Liu L, Ichimura K, *et al.* Amplification and overexpression of the MDM2 gene in a subset of human malignant gliomas without p53 mutations. *Cancer Res* 1993; 53:2736–9.

18 He J, Reifenberger G, Liu L, *et al.* Analysis of glioma cell lines for amplification and overexpression of MDM2. *Genes Chromosom Cancer* 1994; 11:91–6.

19 Ebert M, Yokoyama M, Kobrin MS, *et al.* Increased MDM2 expression and immunoreactivity in human pancreatic ductal adenocarcinoma. *Int J Oncol* 1994; 5:1279–84.

20 Okita S, Tsutsumi M, Onji M, *et al.* p53 mutation without allelic loss and absence of mdm-2 amplification in a transplantable hamster pancreatic ductal adenocarcinoma and derived cell lines but not primary ductal adenocarcinomas in hamsters. *Mol Carcinogenesis* 1995; 13:266–71.

21 Landanyi M, Cha C, Lewis R, *et al.* MDM2 gene amplification in metastatic osteosarcoma. *Cancer Res* 1993; 53:16–18.

22 Lianes P, Orlow I, Zhang Z-F, *et al.* Altered patterns of MDM2 and TP53 expression in human bladder cancer. *J Natl Cancer Inst* 1994; 86:1325–30.

23 Tomonori H, Kinoshita H, Yamada H, *et al.* Oncogene amplification in urothelial cancers with p53 gene mutation or MDM2 amplification. *J Natl Cancer Inst* 1994; 86:1331–5.

24 Quesnel B, Preudhomme C, Oscier D, *et al.* Overexpression of the MDM2 gene is found in some cases of hematological malignancies. *Br J Hematol* 1994; 88:415–18.

25 Chilosi M, Doglioni C, Menestrina F, *et al.* Abnormal expression of the p53 binding protein MDM2 in Hodgkin's disease. *Blood* 1994; 84:4295–300.

26 Finnegan MDM, Goepel JR, Royds J, *et al.* Elevated levels of MDM-2 and p53 expression are associated with high grade non-Hodgkin's lymphomas. *Cancer Letters* 1994; 86:215–21.

27 Selter H, Amela-Neuschwander S, Villena-Heinsen C, *et al.* Antibodies against murine double minute-2 (mdm2) in sera of patients with various gynaecological diseases. *Cancer Lett* 1995; 96:111–15.

28 Corvi R, Savelyeva L, Breit S, *et al.* Non-syntenic amplification of MDM2 and MYCN in human neuroblastoma. *Oncogene* 1995; 10:1081–6.

29 Riou G, Barrois M, Prost S, *et al.* The p53 and mdm-2 genes in human testicular germ-cell tumors. *Mol Carcinogenesis* 1995; 12:124–31.

30 Oliner JD, Kinzler KW, Meltzer PS, *et al.* Amplification of a gene encoding a p53-associated protein in human sarcomas. *Nature* 1992; 358:80–6.

31 Radinsky R, Kraemer PM, Raines MA, *et al.* Amplification and rearrangement of the Kirsten ras oncogene in virus-transformed BALB/c 3T3 cells during malignant tumor progression. *Proc Natl Acad Sci USA* 1987; 34:5143–7.

32 Pollock RE, Lang A, Luo J, *et al.* Soft tissue sarcoma metastasis from clonal expansion of p53 mutated tumor cells. *Oncogene* 1996; 12:2035–9.

33 Adesina AM, Nalbantoglu J, Cavenee WK. p53 gene mutation and mdm2 gene amplification are uncommon in medulloblastoma. *Cancer Res* 1994; 54:5649–51.

34 Poremba C, Yandell DW, Metze D, *et al.* Immunohistochemical detection of p53 in melanomas with rare p53 gene mutations is associated with mdm-2 overexpression. *Oncol Res* 1995; 7:331–9.

35 Kovar H, Auinger A, Jug G, *et al.* Narrow spectrum of infrequent p53 mutations and absence of MDM2 amplification in Ewing tumours. *Oncogene* 1993; 8:2683–90.

36 Kessis TD, Slebos RJ, Han SM, *et al.* p53 gene mutations and MDM2 amplification are uncommon in primary carcinomas of the uterine cervix. *Am J Pathol* 1993; 143:1398–405.

37 Preudhomme C, Quesnei B, Vachee A, *et al.* Absence of amplification of MDM2 gene, a regulator of p53 function, in myelodysplastic syndromes. *Leukemia* 1993; 7:1291–3.

38 Waber PG, Chen J, Nisen PD. Infrequency of MDM2 gene amplification in pediatric solid tumors and lack of association with p53 mutations in adult squamous cell carcinomas. *Cancer Res* 1993; 53:6028–30.

39 Esteve A, Lehman T, Jiang W, *et al.* Correlation of p53 mutations with epidermal growth factor receptor overexpression and absence of mdm2 amplification in human esophageal carcinomas. *Mol Carcinogenesis* 1993; 8:306–11.

40 Landers JE, Haines Ds, Strauss JF III, *et al.* Enhanced translation: a novel mechanism of mdm2 oncogene overexpression identified in human tumor cells. *Oncogene* 1994; 9:2745–50.

41 Olson DC, Marechal V, Momand J, *et al.* Identification and characterization of multiple mdm-2 proteins and mdm-2-p53 proteins. *Oncogene* 1993; 8:2353–60.

42 Gudas JM, Nguyen H, Klein RC, *et al.* Differential expression of multiple MDM2 messenger RNAs and proteins in normal and tumorigenic breast epithelial cells. *Clin Cancer Res* 1995; 1:71–80.

43 Oliner JD, Pietenpol JA, Thiagalingam S, *et al.* Oncoprotein MDM2 conceals the activation domain of tumour suppressor p53. *Nature* 1993; 362:857–60.

44 Leach FS, Tokino T, Meltzer P, *et al.* p53 mutation and MDM2 amplification in human soft tissue sarcomas. *Cancer Res* 1993; 53:2231–4.

45 Cordon-Cardo C, Latres E, Drobnjak M, *et al.* Molecular abnormalities of mdm2 and p53 genes in adult soft tissue sarcomas. *Cancer Res* 1994; 54:794–9.

46 Florenes VA, Maelandsmo GM, Forus A, *et al.* MDM2 gene amplification and transcript levels in human sarcomas: relationship to TP53 gene status. *J Natl Cancer Inst* 1994; 86:1297–1302.
